# Association Between Carotid Artery Perivascular Fat Density and Intraplaque Hemorrhage

**DOI:** 10.3389/fcvm.2021.735794

**Published:** 2021-09-20

**Authors:** Shuai Zhang, Hui Gu, Xinxin Yu, Bing Kang, Xianshun Yuan, Ximing Wang

**Affiliations:** ^1^School of Medicine, Shandong First Medical University, Jinan, China; ^2^Shandong Provincial Hospital Affliated to Shandong First Medical University, Jinan, China

**Keywords:** atherosclerosis, carotid artery, intraplaque hemorrhage, perivascular fat, inflammation

## Abstract

**Objectives:** Perivascular adipose tissue plays a key role in atherosclerosis, but its effects on the composition of carotid atherosclerotic plaques are unknown. This study aimed to investigate the association between inflammatory carotid artery and intraplaque hemorrhage (IPH) in the carotid artery.

**Methods:** This is a single-center retrospective study. Carotid inflammation was assessed by perivascular fat density (PFD) in 72 participants (mean age, 65.1 years; 56 men) who underwent both computed tomography angiography (CTA) and magnetic resonance imaging (MRI) within 2 weeks. The presence of IPH was assessed with MRI. Carotid stenosis, maximum plaque thickness, calcification, and ulceration were evaluated through CTA. The association between PFD and the occurrence of IPH was studied using generalized estimating equations analysis.

**Results:** Of 156 plaques, 72 plaques (46.2%) had IPH. Plaques with IPH showed higher PFD than those without [−41.4 ± 3.9 vs. −55.8 ± 6.5 Hounsfield unit (HU); *p* < 0.001]. After age, calcification, degree of stenosis, maximum plaque thickness, and ulceration were adjusted for, PFD (OR, 1.96; 95% CI, 1.41–2.73; *p* < 0.001) was found to be strongly associated with the presence of IPH.

**Conclusions:** A higher PFD is associated with the presence of IPH in the carotid artery. These findings may provide a novel marker to identify carotid IPH and risk stratification.

## Introduction

Carotid atherosclerotic plaque is a major cause of cerebrovascular disease worldwide (1, 2). In people aged 30–79 years in 2020, the global prevalence of carotid plaque was estimated to be 21.1% (3). Recent studies have found that the composition of atherosclerotic plaque could define the severity of carotid atherosclerosis beyond vascular stenosis, particularly intraplaque hemorrhage (IPH) (4, 5). It has been proved that IPH can promote plaque progression and instability, thereby increasing the risk of occurrence and recurrence of cerebrovascular events (6). Therefore, understanding the pathology of IPH in clinical practice is important, especially after magnetic resonance imaging (MRI) emerged as a non-invasive image modality for IPH detection (7).

To date, many studies think that IPH is caused by the rupture of immature neoangiogenesis (8, 9). The pathologically impaired neovessels can result from vascular inflammation and oxidative stress (9). Perivascular adipose tissue (PVAT) can carry inflammatory components that increase vascular oxidative and vascular inflammation, suggesting PVAT may play a role in the occurrence of IPH (10). However, the specific participation of PVAT in the mechanism of IPH remains unclear. Most currently, the function of PVAT in vascular disease pathogeny has been well-accepted and supported by clinical evidence (11). In addition, an increase in perivascular fat density (PFD) is closely related to PVAT inflammatory changes (12). Saba et al. (13) have recently reported that the carotid PFD that is evaluated by computed tomography angiography (CTA) is linked to contrast plaque enhancement, which is associated with plaque inflammation. An additional study found that the carotid PFD as a marker of vulnerable plaques is closely linked to cerebrovascular ischemic events (14). To the best of our knowledge, few studies have investigated the relationship between carotid PFD and the presence of IPH.

In clinical practice, antithrombotic treatment is used to prevent the risk from IPH, including subsequent cardiovascular events caused (15). However, the recent findings have shown that the use of antithrombotic treatment increases the frequency of IPH in carotid plaques (16). To avoid this paradoxical situation, studying the relationship between pericarotid fat inflammation and IPH and thus proposing a theoretical basis for novel treatment are warranted.

This study aimed to investigate the association between carotid inflammatory, as assessed by PFD, and the presence of IPH in the carotid artery.

## Materials and Methods

### Study Population

Institutional review board approval was obtained for all study procedures, and informed consent was waived because of the retrospective nature of the study. This is a single-center retrospective study. We screened consecutive patients who underwent both CTA and high-resolution vessel wall MRI examinations for suspected atherosclerotic disease of the carotid arteries from January 2018 to December 2020 at Shandong Provincial Hospital Affiliated to Shandong First Medical University. Most patients are examined because of symptoms, such as stroke. The rest of the patients were asymptomatic, and the abnormalities were accidentally found by ultrasonography. The inclusion criterion was that the interval between the two imaging examinations was within 2 weeks. Exclusions criteria were as follows: (1) diseases other than atherosclerotic disease (i.e., dissection); (2) history of carotid endarterectomy and stenting; and (3) CTA and magnetic resonance (MR) images with poor quality. Demographic and clinical data including age, sex, body mass index (BMI), hypertension, hyperlipidemia, diabetes, smoking, coronary artery disease (CAD), antihypertension use, statin utilization, and antiplatelet use were collected from medical record. If patients had a transient ischemic attack or stroke occurring in the vascular territory supplied by the index carotid artery, plaques in that carotid vascular were considered symptomatic. If plaques are not in that vascular or in the vascular of asymptomatic patients, they were considered asymptomatic. Patients were considered asymptomatic if they had no cerebrovascular symptoms in the past 6 months.

### MRI

The high-resolution MRI was performed on a whole-body 3.0-T MR scanner (Ingenia, Philips Healthcare, Best, the Netherlands) with a standard 64-channel head–neck coil. The high-resolution vessel wall MRI protocol was employed with the following parameters: for three-dimensional time-of-flight (3D TOF), fast field echo (FFE), repeat time (TR)/echo time (TE) = 20 ms/3.27 ms, field of view (FOV) = 230 mm × 230 mm, matrix = 256 × 256, and slice thickness = 1 mm; for two-dimensional (2D) T2-weighed imaging, turbo spin echo (TSE), TR/TE = 1,000 ms/26 ms, FOV = 140 mm × 140 mm, matrix = 256 × 256, and slice thickness = 2 mm; for 2D T1-weighted imaging, TSE, TR/TE = 2,500 ms/60 ms, FOV = 140 mm × 140 mm, matrix = 256 × 256, and slice thickness = 2 mm; and for magnetization-prepared rapid acquisition gradient echo (MPRAGE), FFE, TR/TE = 8.8 ms/5.3 ms, flip angle = 15°, FOV = 140 mm × 140 mm, matrix = 256 × 256, and slice thickness = 1 mm.

### CTA Protocol

CTA examination was performed on a third-generation dual-source CT scanner (SOMATOM Force; Siemens Healthineers, Erlangen, Germany). CTA studies were obtained in a helical scanning mode with coverage from the aortic arch to skull vertex. A 60- to 80-ml volume of contrast media (Omnipaque-350, GE Healthcare) was injected at the speed of 4 ml/s, followed by 40 ml of saline flush, using a power injector. Bolus tracking was used to trigger the acquisition 5 s after an attenuation threshold of 100 Hounsfield units (HU) was reached into the aortic arch. The carotid CTA scanning parameters were as follows: tube voltage of 100 kV, pitch of 1.0, reconstructed slice thickness of 0.5 mm, reconstructed slice interval of 0.5 mm, and rotation time of 350 ms.

### Image Analysis

All images were independently evaluated by two radiologists with more than 10 years' experience in vascular imaging, both of whom were blinded to patient information, with any disagreement in assessment being resolved by consensus.

The carotid plaque is defined as a vessel wall thickness of more than 1.5 mm or more encroaching into the lumen or at least 0.5 mm or 50% compared with the surrounding carotid thickness values (1, 3). Carotid IPH was defined by MPRAGE-positive plaque with at least one voxel, demonstrating 1.5 times higher signal intensity relative to adjacent sternocleidomastoid muscle in MRI (17). The kappa values of the IPH parameters within inter-observer and intra-observer were 0.91 (95 CI%, 0.263–1.557) and 0.97 (95 CI%, 0.935–1.005), respectively.

The measurements of CTA markers including the presence of calcification, degree of luminal stenosis, maximum plaque thickness, and occurrence of ulceration were obtained by using post-processing workstation (syngo.via, Siemens Force, Germany). The degree of stenosis was determined in accordance with the North American Symptomatic Carotid Endarterectomy Trial (NASCET) criteria on CTA (18). Plaque ulceration was defined as the presence of at least 2 mm of contrast media outpouching into the plaque on any single plane (19).

**Figure 1:**
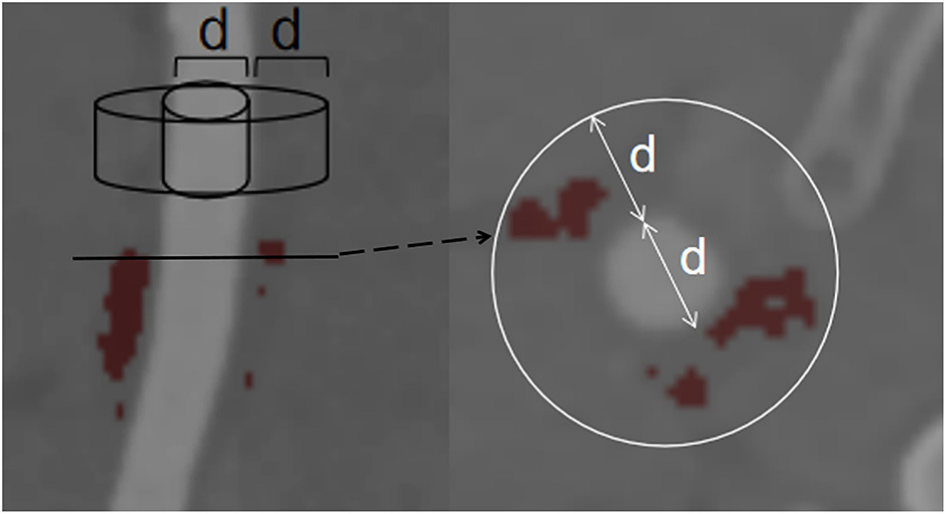
Example of PVAT phenotyping around the carotid artery. Perivascular fat was defined as fat within a radial distance equal to the diameter (d) of the vessel. PVAT, perivascular adipose tissue.

**Figure 2:**
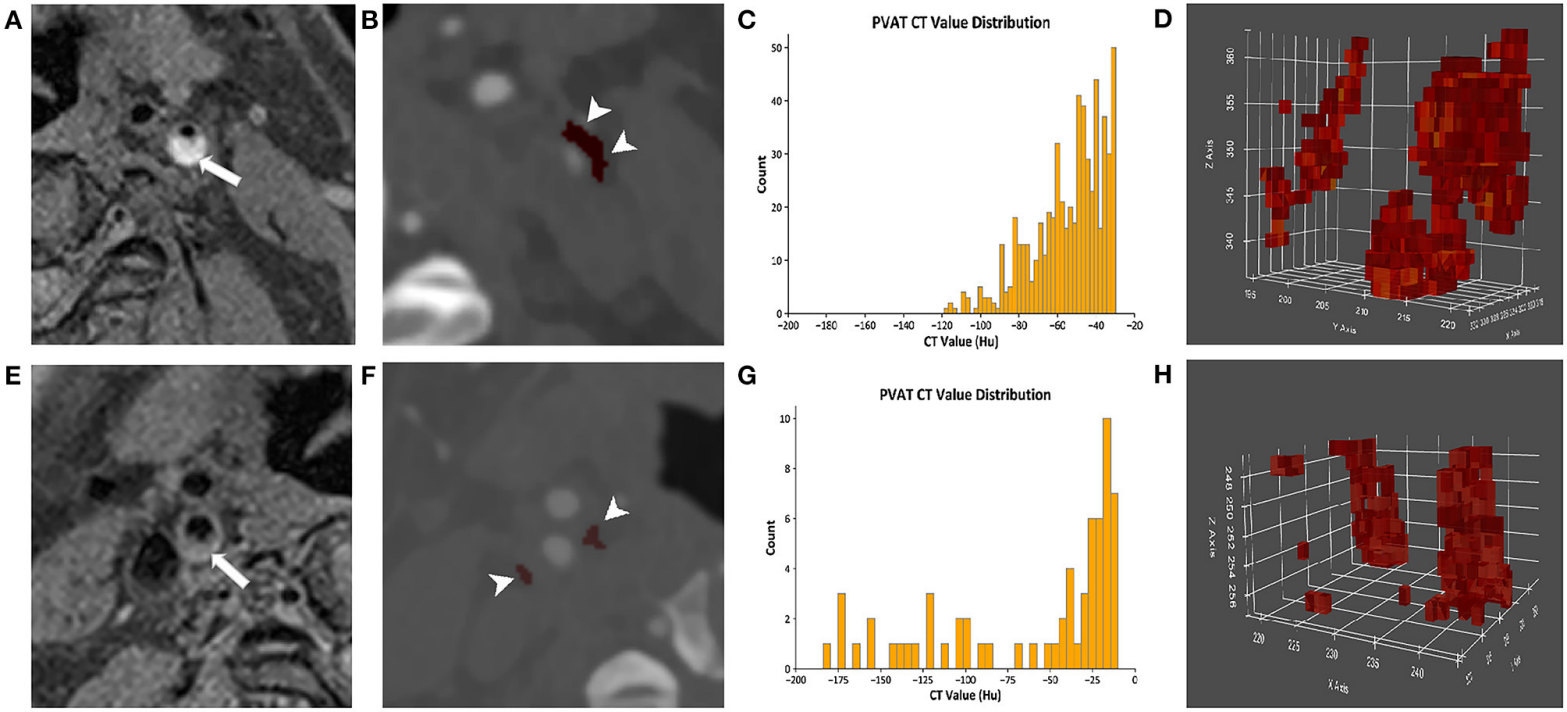
The analysis of the PVAT of plaques with and without IPH. **(A–D)** Come from the same patient. **(A)** A presence of IPH (arrow) was identified on MRI. **(B)** The PVAT (arrowhead) surrounding carotid plaque with IPH was visualized on CTA by red pixels. **(C)** The histogram of PVAT density of plaque with IPH was shown. **(D)** The pixel diagram of PVAT density of plaque with IPH was shown. **(E–H)** Come from the same patient. **(E)** The plaques without IPH (arrow) were identified on MRI. **(F)** The PVAT (arrowhead) surrounding carotid plaque without IPH was visualized on CTA by red pixels. **(G)** The histogram of PVAT density of plaque without IPH was shown. **(H)** The pixel diagram of PVAT density of plaque without IPH was shown. PVAT, perivascular adipose tissue; IPH, intraplaque hemorrhage.

The density of the perivascular fat surrounding the carotid plaque was analyzed in a dedicated software (Perivascular Fat Analysis Tool, Shukun Technology, Beijing, China). Perivascular fat was defined as the adipose tissue surrounding the carotid artery, whose radial distance from the outer vessel wall is equal to the diameter of the vessel (Figure 1) (11). We adapted an established approach used in the coronary arteries and measured the density of the perivascular fat in a semi-automated manner by tracking the contour of vascular stenosis (11). PFD was determined by quantifying the weighed perivascular fat attenuation after adjusting for technical parameters on the basis of the attenuation histogram of perivascular fat within the range −190 to −30 HU. All fat density measurements are reported in HU. The analysis of the PVAT of plaques with and without IPH is shown in Figure 2.

### Statistical Analysis

Continuous variables are described as mean ± standard deviation (SD), and categorical variables are presented as a percentage. Clinical information and carotid plaque characteristics were compared between plaques with and without IPH by using *t*-test and χ^2^ test. Carotid plaque characteristics were compared between plaques with and without symptoms by using Student's *t*-test and χ^2^ test. Correlation coefficients were calculated between the maximum plaque thickness and PFD by Pearson analysis. Correlation analysis was assessed between the ulceration, calcification, and PFD by *t*-test. Logistic regression analysis with generalized estimating equation correction was used to calculate the odds ratio (OR) and corresponding 95% confidence interval (CI) of carotid PFD in discriminating the presence of IPH in the carotid artery. The association between PFD and IPH was analyzed into three models: model 1, adjusted for age; model 2, adjusted for age, calcification, degree of stenosis, maximum plaque thickness, and ulceration; model 3, adjusted for age, BMI, hypertension, hyperlipidemia, diabetes, calcification, degree of stenosis, maximum plaque thickness, and ulceration. Statistical significance was considered at *p* < 0.05. All statistical analyses were performed by using SPSS 22.0 (IBM, Chicago, IL).

## Results

Of 101 subjects who underwent both CTA and high-resolution MRI, 29 were excluded due to normal carotid imaging (*n* = 8), carotid dissection (*n* = 3), poor image quality (*n* = 12), and history of stenting (*n* = 6). Of 72 eligible patients (mean age, 65.1 ± 9.1 years), 56 (77.8%) were male, 48 (66.7%) had hypertension, 34 (47.2%) had hyperlipidemia, 20 (27.8%) had diabetes, 36 (50%) had smoke, and 26 (36.1%) had CAD. The majority was on medical therapy (47.2% on antihypertension, 47.2% on statin, and 56.9% on antiplatelet). The demographic and clinical characteristics are summarized in Table 1.

**Table 1:** Clinical characteristics of the study population (*n* = 72).

	**Mean ± SD or *n* (%)**
Age, years	65.1 ± 9.1
Sex, male	56 (77.8)
BMI, kg/m^2^	26.2 ± 1.7
Hypertension	48 (66.7)
Hyperlipidemia	34 (47.2)
Diabetes	20 (27.8)
Smoking	36 (50.0)
CAD	26 (36.1)
Antihypertension use	34 (47.2)
Statin use	34 (47.2)
Antiplatelet use	41 (56.9)

*BMI, body mass index; CAD, coronary artery disease*.

In this population, 156 plaques were found in the carotid arteries. Of all 156 plaques, 72 (46.2%) had IPH, 118 (75.6%) had calcification, and 10 (6.4%) had ulceration. As shown in Table 2, compared with the carotid plaques without IPH, those with IPH tended to be older (70.8 ± 3.9 vs. 62.4 ± 7.4 years; *p* < 0.001). No significant difference was founded in other clinical information between plaques with and without IPH (*p* > 0.05). Compared with the carotid plaques without IPH, those with IPH showed a higher prevalence of ulceration (12.5% vs. 1.2%; *p* = 0.004), greater stenosis (43.9 ± 21.2% vs. 34.5 ± 16.8%; *p* = 0.002), and greater maximum plaque thickness (3.9 ± 1.5 vs. 2.9 ± 1.0 mm; *p* < 0.001). Calcification was frequently present in the plaques without IPH compared with those with IPH (66.7 vs. 83.3%; *p* = 0.016). The carotid plaques with IPH had higher PFD (−41.4 ± 3.9 vs. −55.8 ± 6.5 HU; *p* < 0.001) than those without IPH. In this population, 47 patients had symptoms, including 22 patients with stroke and 25 patients with transient ischemic attack. Of all 156 plaques, there were 50 plaques without symptoms and 106 plaques with symptoms. Compared with carotid plaques without symptoms, those with symptoms had greater maximum plaque thickness (3.6 ± 1.4 vs. 2.9 ± 1.1 mm; *p* < 0.001), higher PFD (−33.5 ± 11.6 vs. −54.1 ± 8.9 HU; *p* < 0.001), and higher prevalence of IPH (65.1 vs. 4%; *p* < 0.001).

**Table 2:** Comparison of clinical risk factors and CTA characteristics between plaques with and without IPH (*n* = 156).

	**With IPH (*n* = 72)**	**Without IPH (*n* = 84)**	***p*-value**
Age, years	70.8 ± 3.9	62.4 ± 7.4	<0.001
Sex, male	59 (81.9)	68 (81.0)	0.874
BMI, kg/m^2^	26.3 ± 1.6	26.3 ± 1.8	0.963
Hypertension	50 (69.4)	63 (75.0)	0.439
Hyperlipidemia	38 (52.8)	39 (46.4)	0.429
Diabetes	15 (20.8)	21 (25.0)	0.538
Smoking	36 (50.0)	50 (59.5)	0.233
CAD	32 (44.4)	33 (39.3)	0.515
Antihypertension use	40 (55.6)	44 (52.4)	0.692
Statin use	30 (41.7)	42 (50.0)	0.372
Antiplatelet use	32 (44.4)	50 (59.5)	0.060
Calcification	48 (66.7)	70 (83.3)	0.016
Degree of luminal stenosis, %	43.9 ± 21.2	34.5 ± 16.8	0.002
Maximum plaque thickness, mm	3.9 ± 1.5	2.9 ± 1.0	<0.001
Ulceration	9 (12.5)	1 (1.2)	0.004
PFD, HU	−41.4 ± 3.9	−55.8 ± 6.5	<0.001

*BMI, body mass index; CAD, coronary artery disease; IPH, intraplaque hemorrhage, PFD, pericarotid fat density*.

In addition, there was a statistically significant positive correlation between maximum plaque thickness and PFD (*r* = 0.292, *p* < 0.001). We found increased PFD around the plaques without calcification compared with the plaques with calcification (*p* < 0.05). And there was no obvious differences in PFD around plaques with or without ulceration (*p* > 0.05).

In univariate logistic regression analysis, the PFD (OR, 1.70; 95% CI, 1.43–2.04; *p* < 0.001) was found to be strongly associated with the presence of IPH. Multivariate logistic regression revealed that the association between PDF (OR, 1.64; 95% CI, 1.36–1.97; *p* < 0.001) and the occurrence of IPH remained statistically significant, after adjusting for age (model 1). After calcification, degree of stenosis, maximum plaque thickness, and ulceration (model 2) were further adjusted for, the association of PFD (OR, 1.96; 95% CI, 1.41–2.73; *p* < 0.001) with IPH remained significant. After age, BMI, hypertension, hyperlipidemia, diabetes, calcification, degree of stenosis, maximum plaque thickness, and ulceration (model 3) were adjusted for, the association of PFD (OR, 2.33; 95% CI, 1.46–3.72; *p* < 0.001) with IPH remained significant (Table 3).

**Table 3:** Association between PFD and presence of IPH.

	**IPH**							
	**Univariate regression**		**Model 1**		**Model 2**		**Model 3**	
	**OR (95% CI)**	***p*-value**	**OR (95% CI)**	***p*-value**	**OR (95% CI)**	***p*-value**	**OR (95% CI)**	***p*-value**
PFD	1.70 (1.43–2.04)	<0.001	1.64 (1.36–1.97)	<0.001	1.96 (1.41–2.73)	<0.001	2.33 (1.46–3.72)	<0.001

*Model 1: adjusted for age. Model 2: adjusted for age, calcification, ulceration, degree of luminal stenosis, and maximum plaque thickness. Model 3: adjusted for BMI, hypertension, hyperlipidemia, diabetes, calcification, degree of stenosis, maximum plaque thickness, and ulceration. OR, odds ratio; CI, confidence interval; BMI, body mass index; PFD, pericarotid fat density*.

We also performed the same patient analysis comparing pericarotid fat density differences between the carotid arteries on the contralateral and same sides. We found that the PFD around the plaques with IPH was higher than that around the contralateral plaques without IPH (−35.5 ± 2.4 vs. −55.6 ± 0.6 HU; *p* < 0.05). In addition, there was no obvious differences in PFD between the left carotid plaques with IPH and contralateral plaques with IPH (−29.8 ± 7.4 vs. −27.6 ± 8.2 HU; *p* > 0.05) and between the left carotid plaques without IPH and contralateral plaques without IPH (−47.6 ± 7.3 vs. −49.4 ± 5.6 HU; *p* > 0.05). In one vessel, the carotid plaques with IPH had higher PFD than those without IPH (−34.9 ± 2.0 vs. −48.7 ± 5.5 HU; *p* < 0.05).

## Discussion

This study investigated the relationship between the PFD and IPH in carotid plaques. We found that the PFD was significantly associated with the presence of IPH before and after adjusting for confounding factors. Our findings indicate that the PFD might be independent indicators for IPH in carotid plaques.

In this study, we found that the plaques with IPH had greater stenosis and maximum plaque thickness than those without IPH. This finding can be ascribed to the fact that IPH can induce inflammation to accelerate plaque growth and thus result in lumen narrowing and plaque thickening (20). These findings are consistent with previous studies (21, 22). Eisenmenger et al. (21) reported that carotid stenosis is worse in plaques with IPH than in those without (53.5 vs. 24.9%; *p* < 0.001) and that the maximum plaque thickness is higher in groups positive for IPH than in groups negative for IPH (5.93 vs. 3.42; *p* < 0.001). The strong association between ulceration and IPH in the present study agrees with previous findings (19, 23). A recent study has shown a significantly higher prevalence of ulceration (87.3 vs. 31.8%; *p* < 0.001) in plaques with IPH than in those without (23). In the present study, the carotid plaques without IPH showed a higher prevalence of calcification than those with IPH. This finding is consistent with those of previous studies (24, 25). Some researchers found that asymptomatic patients have more calcium deposits in carotid plaques than symptomatic ones, supporting that calcification is a protective factor (24). However, the association between calcification and IPH is not uniform in several studies (22, 26). Lin et al. (22) reported that calcification is usually accompanied by IPH compared to those without (87.5 vs. 55.9%; *p* < 0.05). Microcalcifications are difficult to detect with MRI in their studies. The CTA that we used has higher accuracy in detecting calcification. The different examination modality and the number of patients may account for the difference. Moreover, in recent studies about carotid PFD, several researchers reported the association between contrast plaque enhancement and cerebrovascular ischemic events with PFD (13, 14). However, they did not use software specifically designed to measure carotid PFD. To our knowledge, this study is the first to investigate the association between carotid PFD and the presence of IPH.

The relationship between the PVAT and IPH is complex and likely bidirectional. To date, the pathophysiology of IPH is not completely clear, but the prevailing viewpoints are that IPH develops by rupture of the immature neovessels (8, 9). A wide variety of bioactive molecules from PVAT can diffuse directly into the vascular wall in a paracrine way, thus directly affecting its biology, including inflammation and oxidative stress (10). In advanced atherosclerotic diseases, inflammation and oxidative stress can lead to the secretion of the vascular endothelial growth factor (VEGF) and thus increase immature neoangiogenesis (9). These neovessels, which lack smooth muscle cells and endothelial gap junctions, are pathological and impaired and thus are prone to rupture (27). The rupture of these neovessels results in the formation of IPH. Conversely, some studies have recently shown that the inflamed human vascular wall caused by IPH also releases inflammatory cytokines, which also disseminate into the perivascular space, triggering PVAT inflammatory changes (28, 29). These PVAT changes can be assessed by differences in the fat attenuation as measured by HU on CT. In addition, increased PFD is closely associated with histopathological markers of inflammation (30). Therefore, the PFD is closely associated with the occurrence of IPH.

The role of PVAT in the pathogenesis of vascular diseases is presently well-accepted and supported by translational and clinical evidence (11, 31, 32). Oikonomou et al. (11) reported that the perivascular fat attenuation index, which can capture coronary inflammation, can predict and stratify cardiovascular risk. Thus, this index can serve as a guide for early targeted prevention. Systemic markers of inflammation, including C-reaction protein or proinflammatory cytokines, have been associated with cardiovascular risk prediction, but they are often driven by other inflammatory conditions and unable to pinpoint local inflammation-induced changes (29, 33). Conversely, measuring PFD in CTA is helpful in identifying localized perivascular inflammation (34, 35). Positron emission tomography (PET) imaging is the gold standard in evaluating PVAT inflammation assessed by FDG uptake, but its use is limited by its high exposure, high cost, and low clinical availability (36). Recent studies have reported that local coronary inflammation associated with vulnerable plaques can be detected by using standard non-invasive CTA modality, providing information similar to ^18^F-NaF PET-CT (37). In addition, CTA is more cost-effective than PET.

IPH, a compositional characteristics of vulnerable plaques, contributes to plaque progression and rupture, thus increasing the risk of cerebrovascular ischemic events (5, 6, 38). Recently, several studies have shown that the use of antithrombotic treatment is associated with a higher frequency of IPH in the carotid atherosclerotic plaques (16, 39). These findings seem to contradict current knowledge because antithrombotic treatment is used to reduce the risk from IPH, including subsequent cardiovascular events caused (15, 40). In the present study, we found the association between IPH and carotid inflammation, providing a theoretical basis for new targeted anti-inflammatory therapy. At present, considerable studies have focused on preventing atherosclerosis by intervening in PVAT (41, 42). Therefore, PVAT may be emerging as the therapeutic target for IPH.

Several limitations of our study should be noted. First, this is a retrospective study, and the cause–effect of perivascular inflammation and IPH cannot be determined. Future prospective studies could test this causality relationship. Second, histological validation of IPH remains the goal standard, but this was not available in this study. However, MRI is currently the best *in vivo* modality for IPH, and the technique used in this study has previously been validated in comparison with histology (7). Third, the range of PFD measurements was uncertain, and our study adapted an established method used in coronary arteries (11). Finally, we cannot obtain clinical data on specific measurements, such as blood pressure and laboratory data from clinical records in our study. The patients were indeed hypertensive or hyperlipidemic, but specific measurements were not recorded in the medical records for some reasons. For example, the laboratory data of several patients were done in other hospital, and the clinical doctors only recorded whether they had hypertension or hyperlipidemia and did not record specific measurements in the medical records.

In conclusion, our study shows the PFD is independently associated with IPH in the carotid artery, suggesting that PVAT may play a key role in the occurrence of IPH. Our findings may provide a novel marker to identify carotid IPH and risk stratification.

## Data Availability Statement

The original contributions presented in the study are included in the article/supplementary material, further inquiries can be directed to the corresponding author/s.

## Ethics Statement

The studies involving human participants were reviewed and approved by Medical Ethics Committee of Shandong Provincial Hospital. Written informed consent for participation was not required for this study in accordance with the national legislation and the institutional requirements. Written informed consent was not obtained from the individual(s) for the publication of any potentially identifiable images or data included in this article.

## Author Contributions

SZ and BK have substantial contributions to the conception or design of the work and to the acquisition, analysis, and interpretation of data for the work. HG, XYu, and XYua have substantial contributions to drafting the work or revising it critically for important intellectual content. XW has substantial contributions to the final approval of the version to be published and agreed to be accountable for all aspects of the work in ensuring that questions related to the accuracy or integrity of any part of the work are appropriately investigated and resolved. All authors contributed to the article and approved the submitted version.

## Funding

The present study was supported by the National Natural Science Foundation of China (grant nos. 8187354, 81571672, and 81371548) and Academic Promotion Programme of Shandong First Medical University (grant no. 2019QL023).

## Conflict of Interest

The authors declare that the research was conducted in the absence of any commercial or financial relationships that could be construed as a potential conflict of interest.

## Publisher's Note

All claims expressed in this article are solely those of the authors and do not necessarily represent those of their affiliated organizations, or those of the publisher, the editors and the reviewers. Any product that may be evaluated in this article, or claim that may be made by its manufacturer, is not guaranteed or endorsed by the publisher.
